# The changes in family functioning and family happiness during the COVID-19 pandemic: The situation in Thailand

**DOI:** 10.3389/fpubh.2022.1055819

**Published:** 2022-12-22

**Authors:** Nida Limsuwan, Thanavadee Prachason, Pattarabhorn Wisajun

**Affiliations:** Department of Psychiatry, Faculty of Medicine, Ramathibodi Hospital, Mahidol University, Bangkok, Thailand

**Keywords:** family functioning, family happiness, COVID-19, coronavirus, pandemic

## Abstract

**Background:**

The effects of the COVID-19 pandemic on family well-being and functioning were generally a concern for healthcare providers in many countries.

**Objectives:**

To explore the changes in family functioning and family happiness during the pandemic in Thailand and to investigate factors associated with the changes in family happiness.

**Methods:**

This was a cross-sectional study conducted between November and December 2021. Online questionnaires regarding family functioning, family happiness, domestic violence, and COVID-19-related experiences were used.

**Results:**

A total of 485 participants were included in this study. The perceived family happiness slightly decreased from 8.19 (pre-pandemic score) to 7.62 (post-pandemic score). In contrast, the general family functioning (SCORE-15 index), strength, and communication subscale scores after the onset of the COVID-19 pandemic were significantly lower than those of the pre-pandemic period. Moreover, the prevalence of verbal and physical violence significantly reduced during the pandemic. In addition, the change in family functioning was the strongest factor associated with the change in family happiness.

**Conclusion:**

In general, family functioning slightly improved; however, perceived family happiness decreased during the pandemic. In addition, the change in family functioning was the strongest factor associated with the change in family happiness.

## Introduction

The COVID-19 pandemic started in China in late 2019; however, later the World Health Organization (WHO) globally reported more than 500 million confirmed cases and more than 6 million deaths by May 2022 (1). Several preventive measures, including physical distancing, quarantines, lockdowns, school closures, and working from home, were enforced in many countries during the pandemic. It profoundly impacted individuals, families, communities, and healthcare systems from the direct effects of the pandemic and also the indirect effects of measures taken. In addition, several studies demonstrated an increased prevalence of mental health problems such as substance use, feeling of loneliness, and depressive and anxiety disorders (2–5). Moreover, some studies reported an increase in domestic violence and child maltreatment during the COVID-19 pandemic (6–9). As a result, the effects of the COVID-19 pandemic on family well-being and functioning were generally a concern for healthcare providers.

Several studies revealed the association between the COVID-19 situations and the well-being of families; for example, Lee and colleagues (10) reported social determinants of health had direct effects on COVID-19-related stress, family stress, and family discord in North Carolina, USA. Another study from the USA (11) demonstrated a large deterioration in parent and child mental and behavioral health during the first months of the pandemic in children internalizing and externalizing problems and parent depression. The proportion of children rated in the clinical range increased by 2.5 and 4 times in internalizing and externalizing problems, respectively, from pre-pandemic levels. Moreover, an Australian study compared before and during the pandemic datasets regarding family situations and showed higher levels of parenting irritability and lower levels of family-positive expressiveness during the pandemic. However, the evidence for couple verbal conflict was inconclusive according to this study (12). In addition, Zeng and colleagues (13) conducted research on Chinese college students and found that family cohesion was negatively related to stress related to COVID-19. Congruently, a study from South Korea (14) reported that family cohesion had a significant mediating effect on the relationship between positive psychological capital and health promotion behaviors during the COVID-19 pandemic.

In general, family functioning is defined by several dimensions, including the quality of relationships among family members, the effectiveness of family communication and problem-solving, family cohesiveness, and family adaptability (15). Family function possibly indicates family situations, challenges, adjustments, and resilience during abnormal circumstances. Although some studies revealed family situations and well-being during the COVID-19 pandemic, few studies actually focused on using standard measurements that are specific to family functioning assessments. Moreover, the comparisons of family functioning before and during the pandemic are rarely reported in previous studies. In addition, the COVID-19 situations and preventive measures enforced in each country were different. Therefore, this present study aims to explore the changes in family functioning and family happiness before and during the pandemic and to investigate factors associated with the change in family happiness in the Thai population.

## Materials and methods

### Sampling and data collection

This was a cross-sectional study that used an online survey because the Thai government generally asked for cooperation to stay at home when this study was conducted. The survey was conducted between November and December 2021. At that period, the COVID-19 situations in Thailand were serious. There were about 18,400–56,000 confirmed cases and 140–460 deaths weekly (16). The questionnaire was distributed *via* the Internet on the official website and Facebook pages of the Department of Psychiatry, Ramathibodi Hospital. Moreover, the questionnaire link was sent to famous Facebook pages regarding mental health and psychology in Thailand. The inclusion criteria were (1) age ≥ 18 years, (2) informed consent, and (3) ability to understand the questionnaire in Thai.

### Measurements

#### Sociodemographic characteristics and COVID-19-related experiences

The participants were invited to answer questions regarding gender, age, marital status, region, education, occupation, income, and underlying disease. Moreover, they were asked to rate the fear of COVID-19 infection from 0 to 10 and to report if they had any of these COVID-19-related experiences during the pandemic (yes/no): (1) history of being infected with COVID-19, (2) having close persons infected with COVID-19, (3) having close persons died of COVID-19, (4) having quarantine experiences, and (5) having financial problems because of the COVID-19 pandemic. Exposure to each category of experiences was counted as one point, and the number of COVID-19-related experiences (range: 0–5) was summed up to reflect the degree to which a person was affected by the pandemic.

#### Family functioning and family happiness

The 15-item Systemic Clinical Outcome and Routine Evaluation (SCORE-15) is a self-report questionnaire completed by family members aged 12 years and older. It records perceptions of the family from each member and measures both overall functioning and specific aspects of family functioning, including the following three domains: family strengths, difficulties, and communication (17). It includes 15 Likert scale items. The SCORE-15 Thai version was developed following the translation protocol of the SCORE team as previously reported (18). The test–retest reliability was excellent, with an intraclass correlation coefficient of 0.94. The criterion validity study revealed that the effect sizes of score differences between clinical and non-clinical Thai samples were large (0.90–1.85) (18). In addition, the recent study generally exhibited good internal reliability and convergent validity of the SCORE-15 Thai version in the Thai population (19). All available items in each domain were averaged to indicate the domain index (range: 1–5) only if the number of missing items in that domain was no more than one. Only if all three domain indexes were not missing, all available SCORE-15 items were averaged to indicate the SCORE-15 index (range: 1–5). The greater SCORE-15 index reflects poorer family functioning.

In this present study, the participants were invited to answer 2 sets of questions on the SCORE-15 Thai version with different lead-in phrases. For the assessment of family functioning before the pandemic, the lead-in phrase was “please answer the following questions by considering your situations before the COVID-19 epidemic,” and for the assessment of family functioning during the pandemic, the lead-in phrase was “please answer the following questions by considering your situations during the COVID-19 epidemic.” Moreover, three additional questions regarding physical violence, verbal violence, and happiness within the family were added at the end of the SCORE questionnaire. The 0–10 rating scale was used to assess perceived family happiness.

### Ethical standards

This present study's procedures were approved by the Ethics Committee of Ramathibodi Hospital, Mahidol University. The participants were provided an information statement and asked to give online informed consent for the research in accordance with the Declaration of Helsinki. In addition, all data and documents have been anonymized, and the datasets were non-personally identifiable.

### Statistical analysis

Data analyses were performed using Stata version 13.0. Out of the 487 responses, two failed to complete either set of questionnaires regarding pre- or post-pandemic happiness and were thus excluded from further analyses. The sociodemographic characteristics of the included participants and their experiences related to the COVID-19 pandemic were presented using descriptive statistics. The bootstrapping approach was used to determine the significance of inferential statistics and estimate 95% bias-corrected and accelerated bootstrap (BCa) confidence intervals (CIs) of the parameters. More specifically, to determine the change in family atmosphere, the percentages of physical and verbal violence in families before and after the onset of the COVID-19 pandemic were compared using the bootstrap chi-square, and the scores of perceived family happiness and family functioning were compared likewise using bootstrap paired *t*-test. The association between each variable and the changes in family happiness score, generated by subtracting the pre-pandemic happiness scores from the post-pandemic scores, was evaluated using bootstrap unpaired *t*-test for two categorical variables, bootstrap ANOVA for more than two categorical variables, and bootstrap Pearson's correlation for continuous variables. Statistical significance was set at a *P*-value of <0.05. Factors significantly associated with the changes in family happiness in univariate analyses were then included as independent variables in subsequent bootstrap multiple linear regression analyses. All re-samplings were iterated 2,000 times while allowing for sample replacement.

## Results

### Sample characteristics

Of the 485 participants included in this study, the majority were women (*N* = 441, 90.9%). The age ranged from 19 to 67 years (mean = 41.7, SD = 8.62). Most participants had a bachelor's degree (*N* = 247, 50.9%) or above (*N* = 201, 41.5%) as their highest education. Of the participants, two-thirds (*N* = 323) earned more than 40,000 THB/month, whereas 12.6% (*N* = 61) earned < 20,000 THB/month. Regarding COVID-19-related experiences, the average score for fear of infection was 7.4 (range 0–10, SD = 2.3). Of the participants, one-fourth (26.2%) had quarantine experience, but only 12 (2.5%) reported a history of COVID-19 infection. Approximately 37% (*N* = 178) had close persons infected with COVID-19, and 11.0% (*N* = 53) reported having close persons died of COVID-19. Around two-thirds (*N* = 331) reported having financial problems during the COVID-19 pandemic. Overall, most participants experienced one to two COVID-19-related events (Mdn = 1, IQR = 1–2, Table 1).

**Table 1:** Characteristics of the participants.

	**Total**	***N* (%)**
**Demographic characteristics**		
**Age**, Mean (SD)	481	41.7 (8.6)
**Female**	485	441 (90.9)
**Marital status**	484	
Single		120 (24.8)
Married		340 (70.2)
Divorced/widowed		24 (5.0)
**Buddhist**	485	456 (94.0)
**Education**	485	
Below bachelor degree		37 (7.6)
Bachelor degree		247 (50.9)
Above bachelor degree		201 (41.5)
**Work status**	485	
Studying		13 (2.7)
Employed with regular income		277 (57.1)
Employed with non-regular income		96 (19.8)
Unemployed or non-paid working		99 (20.4)
**Monthly income**	484	
< 20,000 THB		61 (12.6)
20,000, 40,000 THB		100 (20.7)
>40,000 THB		323 (66.7)
**Underlying illness or health problems**	485	173 (35.7)
**Experiences related to COVID-19**		
**Fear of infection** (0–10), Mean (SD)	484	7.4 (2.3)
**COVID-19 infection**	485	12 (2.5)
**Close persons infected with COVID-19**	485	178 (36.7)
**Close persons died of COVID-19**	482	53 (11.0)
**Quarantine**	484	127 (26.2)
**Financial problems**	485	331 (68.2)
**Number of COVID-19-related experiences**	481	
None		80 (16.6)
1–2		319 (66.3)
3 or more		82 (17.1)

### Changes in the family atmosphere during the COVID-19 pandemic

The prevalence of physical violence significantly reduced from 14.3% (95% bootstrap CI: 68.2–76.3%) during the pre-COVID-19 pandemic period to 6.2% (95% bootstrap CI: 4.1–8.4%) after the onset of the pandemic (bootstrap *P* = 0.034). The prevalence of verbal violence was far more prevalent than physical violence but similarly decreased from 72.3% (95% bootstrap CI: 68.2–76.3%) to 51.3% (95% bootstrap CI: 46.7–56.0%) (bootstrap *P* = 0.001). The perceived family happiness slightly decreased from 8.19 (pre-pandemic score) to 7.62 (post-pandemic score). In addition, the general family functioning (SCORE-15 index), strength, and communication subscale scores after the onset of the COVID-19 pandemic were significantly lower than those of the pre-pandemic period (all bootstrap *P* < 0.05, Table 2). On the contrary, the difficulty subscale score of family functioning did not significantly change during the pandemic (bootstrap *P* = 0.783, Table 2).

**Table 2:** Family atmosphere and family functioning before and after the COVID-19 pandemic.

	**Mean [95% BCa CI]**		**Bootstrap *P*-value**
	**Before**	**After**	
Verbal violence, *N (%)* [95% bootstrap CI]	349 (72.3) [68.2, 76.3]	249 (51.3) [46.7, 56.0]	0.001
Physical violence, *N (%)* [95% bootstrap CI]	69 (14.3) [11.1, 17.4]	30 (6.2) [4.1, 8.4]	0.034
Perceived family happiness	8.19 [8.01, 8.36]	7.62 [7.43, 7.81]	<0.001
Family functioning index (SCORE-15)	2.22 [2.16, 2.28]	2.18 [2.12, 2.24]	0.007
Strength subscale	1.98 [1.93, 2.05]	1.94 [1.88, 2.01]	0.022
Difficulty subscale	2.40 [2.33, 2.48]	2.40 [2.31, 2.48]	0.783
Communication subscale	2.28 [2.22, 2.35]	2.20 [2.13, 2.27]	<0.001

### Factors associated with changes in family happiness score

Univariate analyses demonstrated that a decrease in happiness score tended to be larger among women compared to men, but the association did not pass the threshold of statistical significance (bootstrap *P* = 0.087). No significant associations between happiness score change, and marital status, religion, education, work status, income, or health problems were found (all bootstrap *P* > 0.05, Table 3). Experiencing a loss of close persons from COVID-19 (bootstrap *P* = 0.052) and having financial problems (bootstrap *P* = 0.036) were associated with a larger decrease in family happiness score but neither the experiences of COVID-19 infection (oneself or close persons) nor the experiences of quarantine were associated with changes in family happiness (Table 3).

**Table 3:** Associations between participants' characteristics and changes in perceived family happiness.

	**Happiness changes Mean [95% BCa CI]**	***P*-value**
**Demographic characteristics**		
**Gender**		
Male	−0.25 [−0.63, 0.10]	0.087
Female	−0.59 [−0.72, 0.47]	
**Marital status**		
Single	−0.49 [−0.72, −0.26]	0.833
Married	−0.59 [−0.75, −0.45]	
Divorced/Widowed	−0.58 [−1.18, −0.08]	
**Religion**		
Buddhism	−0.55 [−0.68, −0.43]	0.478
Non–Buddhism	−0.72 [−1.24, −0.30]	
**Education**		
Below bachelor degree	−1.03 [−1.68, −0.48]	0.348
Bachelor degree	−0.44 [−0.61, −0.27]	
Above bachelor degree	−0.63 [−0.82, −0.46]	
**Work status**		
Studying	−0.69 [−1.20, −0.25]	0.939
Employed with regular income	−0.57 [−0.73, −0.43]	
Employed with non–regular income	−0.55 [−0.83, −0.29]	
Unemployed or non–paid working	−0.53 [−0.88, −0.19]	
**Monthly income**		
<20,000 THB	−0.75 [−1.28, −0.25]	0.151
20,000 to 40,000 THB	−0.92 [−1.24, −0.60]	
> 40,000 THB	−0.42 [−0.54, −0.30]	
**Underlying illness or health problems**		
Yes	−0.58 [−0.76, −0.41]	0.850
No	−0.55 [−0.72, −0.39]	
**Previous violence**		
Yes	−0.41 [−0.78, −0.03]	0.361
No	−0.59 [−0.72, −0.46]	
**Previous verbal violence**		
Yes	−0.54 [−0.69, −0.40]	0.571
No	−0.63 [−0.89, −0.39]	
**Experiences related to COVID−19**		
**COVID−19 infection**		
Yes	−1.17 [−2.22, −0.40]	0.171
No	−0.55 [−0.67, −0.43]	
**Close persons infected with COVID−19**		
Yes	−0.65 [−0.84, −0.46]	0.308
No	−0.51 [−0.67, −0.36]	
**Close persons died of COVID−19**		
Yes	−0.91 [−1.25, −0.56]	0.052
No	−0.53 [−0.66, −0.40]	
**Quarantine**		
Yes	−0.73 [−1.01, −0.48]	0.116
No	−0.50 [−0.63, −0.36]	
**Financial problems**		
Yes	−0.65 [−0.81, −0.49]	0.036
No	– 0.38 [−0.57, −0.20]	

For continuous variables, the change in family happiness was positively correlated with age (rho = 0.12, 95% BCa CI: 0.03 – 0.20, *N* = 481, *P* = 0.007), but negatively correlated with family happiness before the pandemic (rho = −0.22, 95% BCa CI: −0.31 to −0.12, *N* = 485, *P* < 0.001), the number of COVID-19-related experiences (rho = −0.13, 95% BCa CI: −0.22 to −0.05, *N* = 481, *P* = 0.002), and the changes in SCORE-15 scores (rho = −0.34, 95% BCa CI: −0.47 to −0.22, *N* = 479, *P* < 0.001). No significant correlation between the change in family happiness and fear of the infection was observed (rho = −0.05, 95% BCa CI: −0.14–0.05, *N* = 484, *P* = 0.281).

Factors associated with the changes in perceived family happiness in univariate analyses were subsequently tested in multiple linear regression analyses. As shown in Table 4, age was significantly associated with the changes in family happiness in both models (Model 1: Beta = 0.02, bootstrap *SE* = 0.01, *P* = 0.011; Model 2: Beta = 0.02, bootstrap *SE* = 0.01, *P* = 0.018), but sex was not (Model 1: Beta = −0.14, bootstrap *SE* = 0.21, *P* = 0.482; Model 2: Beta = −0.18, bootstrap *SE* = 0.20, *P* = 0.389). Perceived family happiness before the pandemic (Model 1: Beta = −0.20, bootstrap *SE* = 0.04, *P* < 0.001; Model 2: Beta = −0.20, bootstrap *SE* = 0.04, *P* < 0.001) and changes in the SCORE-15 index (Model 1: Beta = −1.60, bootstrap *SE* = 0.24, *P* < 0.001; Model 2: Beta = −1.57, bootstrap *SE* = 0.24, *P* < 0.001) also consistently showed significant negative associations with the changes in family happiness. The experience of financial problems during the pandemic was significantly associated with a 0.29-point reduction of perceived family happiness after controlling for other factors (Model 1: Beta = −0.29, bootstrap *SE* = 0.12, *P* = 0.014), whereas the experience of close persons died of COVID-19 infection only showed a trend of association without reaching the threshold of statistical significance (Model 1: Beta = −0.29, bootstrap *SE* = 0.16, *P* = 0.071). However, each additional exposure to COVID-19-related experiences was associated with a 0.14-point decrease in family happiness scores as shown in Model 2 (Beta = −0.14, bootstrap *SE* = 0.05, *P* = 0.009; Table 4).

**Table 4:** Multiple linear regression of factors associated with changes in perceived family happiness.

	**Model 1**		**Model 2**	
	**Beta [95% BCa CI]**	***P*** **-value**	**Beta [95% BCa CI]**	***P*** **-value**
Age	0.02 [0.004, 0.03]	**0.011**	0.02 [0.003, 0.03]	**0.018**
Female	−0.14 [−0.49, 0.32]	0.482	−0.18 [−0.52, 0.31]	0.389
Family happiness score before the pandemic	−0.20 [−0.29, −0.13]	**<0.001**	−0.20 [−0.29, −0.12]	**<0.001**
Changes in SCORE−15 index	−1.60 [−2.07, −1.13]	**<0.001**	−1.57 [−2.04, −1.13]	**<0.001**
Close persons died of COVID−19	−0.29 [−0.66, 0.01]	0.071		
Financial problems	−0.29 [−0.52, −0.06]	**0.014**		
Number of COVID−19–related experiences			−0.14 [−0.25, −0.04]	**0.009**
Constant	0.70 [−0.09, 1.61]	0.097	0.70 [−0.10, 1.58]	0.100

Statistically significant results (P < 0.05) were presented in bold.

## Discussion

This study aims to explore the changes in family happiness and family functioning after the COVID-19 pandemic and to identify factors associated with the change in family happiness in the Thai general population. Expectedly, we found that perceived family happiness significantly decreased during the pandemic. Surprisingly, the reported prevalence of verbal and physical violence in families during the pandemic period appeared lower than that of the pre-pandemic period, and the overall family functioning was improved during the period, as reflected by the significant reduction of the SCORE-15 scores. The identification of associated factors revealed that those who had close persons who died of COVID-19 or had financial problems showed a larger decrease in perceived family happiness compared to those without these experiences; however, the former issue lost its statistical significance after adjustment for other factors. Interestingly, we found a one-point increase in the SCORE-15 index, which reflects worsening family functioning, was associated with around a 1.6-point decrease in perceived family happiness, whereas each additional exposure to COVID-19-related experiences was associated with only a 0.14-point decrease in family happiness scores.

In general, the COVID-19 pandemic possibly caused many stressful environments, for example, quarantines, social isolation, economic instability, deaths, and loss. These stressors inevitably affected family systems. Unsurprisingly, we found a decrease in perceived family happiness during the COVID-19 pandemic. Several studies revealed an increase in stress and psychological problems in families during the pandemic, especially during lockdown periods (10, 12, 20, 21). For example, Giannotti and colleagues (21) investigated family adjustment to the COVID-19 lockdown in Italy. The results showed that parental stress, especially in mothers, and child externalizing behaviors increased during the lockdown period. In addition, Westrupp and colleagues (12) reported higher rates of parental depression, anxiety, and stress; higher parenting irritability; lower family-positive expressiveness; and higher alcohol consumption in Australia during the COVID-19 pandemic compared to pre-pandemic estimates. However, this study highlighted the importance of family functioning in terms of influences on family happiness over situational factors, such as losing close persons and financial problems. In fact, family functioning *per se* reflects the adaptation of families, which can be measured in several dimensions such as communication and problem-solving ability (22). Therefore, the change in family functioning potentially demonstrated a stronger association with the change in family happiness during the COVID-19 pandemic than other factors that directly represented stressors *per se*.

Focusing on family functioning, we found that general family functioning, strength subscale, and communication subscale of family functioning significantly improved during the COVID-19 pandemic. These results might be explained by the timing of data collection, which was around the late second wave of the COVID-19 outbreak in Thailand. People might have learned how to cope with the outbreak since the first wave; as a result, they might function better in terms of intrapersonal adjustment as well as interpersonal adjustment which is reflected in family functioning. In contrast, Hussong and colleagues (23) reported a decrease in open family communication, parental support of youths, and family satisfaction during the pandemic in the USA, according to child reports, although no changes were found in parent reports of family functioning. In addition, Zeng and colleagues (13) found family cohesion was negatively related to stress consequences among Chinese college students during the pandemic. Moreover, regarding the mechanism by which family cohesion helped decrease stress consequences, they demonstrated that affective empathy had a moderating effect on the stress consequences. However, it was possible that family functioning and stress related to the COVID-19 pandemic had bidirectional relationships. Moreover, Yang and colleagues (24) reported that generalized anxiety disorder and trait anxiety had mediating effects between family functioning and state anxiety. However, other potential mediators and moderators might play important roles in the relationship between family functioning and anxiety/stress related to the COVID-19 pandemic.

Regarding family violence, some studies reported a high prevalence of domestic violence during the COVID-19 pandemic. For example, the community-based survey in India (6), which involved 209 married women aged 18–55 years, revealed the prevalence of domestic violence during the pandemic and lockdown to be 25.8%, and in particular the prevalence of severe domestic violence to be 6.2%. Recently, systematic reviews (25, 26) reported an increase in domestic violence cases all over the world; however, some studies demonstrated the opposite direction. For instance, Halford and colleagues (27) revealed a decrease in domestic abuse (−45%) according to daily counts of recorded crime data from a UK police service by comparing the data in 2020 to the expected rates based on crimes recorded in the previous 4 years. Moreover, McLay (28) reported domestic violence cases collected from the Chicago Police Department slightly decreased from 2,367 cases in March 2019 (pre-pandemic) to 2,251 cases in March 2020 (pandemic). Although these results showed a decrease in the rate of domestic violence, they were more likely to be underreported or have changes in help-seeking behaviors than an actual decline. According to the systematic review by Kourti and colleagues (25), an increase in domestic violence was noted globally during the first week of the lockdown. Surprisingly, we found a significant decrease in both verbal and physical violence in families during the COVID-19 pandemic. However, there was not a complete lockdown in Thailand at the time of this survey, but the government generally asked for cooperation to stay at home due to the serious outbreak. Perhaps living arrangements or home confinement might play an important role in domestic violence. In addition, regarding the COVID-19 situation in Thailand, the time when we conducted this survey was during the late second wave of the epidemic in Thailand. Most people inevitably had to deal with stressful situations and possibly gained their coping skills since the first wave of the COVID-19 epidemic, when there were lockdowns in several regions of Thailand, especially Bangkok.

## Strengths and limitations

The strengths of this study included using the standard assessment of family functioning, which was directly designed to assess both general functioning and specific dimensions of family functioning. In addition, this study was timely conducted during the serious period of the COVID-19 pandemic in Thailand. Moreover, we accounted for and gathered data regarding COVID-19-related experiences, including the history of COVID-19 infection, the experiences of losing close persons, quarantine, and financial problems.

However, this study has several limitations. First, pre-pandemic data were retrospectively gathered; as a result, recall bias was inevitably avoided. Second, family functioning and the pandemic situations were dynamic over time. It was impossible to demonstrate patterns of change over time by using a cross-sectional design. Third, this study probably had low generalizability because the study design was an online survey *via* the links on the official website and educational Facebook pages. Therefore, the majority of the study population were women in middle to upper socioeconomic classes who were easily accessible to the Internet. Further studies should be conducted in a broader variety of populations, including those with lower socioeconomic backgrounds. Finally, the COVID-19 situation in Thailand generally tended to be less severe compared to many countries in Europe and the USA in terms of the prevalence of infection and mortality. Consequently, it was difficult to compare the results of this study to the studies from other countries which encountered different situations, public health systems, and social contexts.

## Conclusion

Regarding the situation in Thailand, the perceived family happiness slightly decreased during the COVID-19 pandemic. In contrast, the prevalence of verbal and physical violence in families significantly reduced. In addition, the results demonstrated that the general family functioning, strength subscale, and communication subscale of family functioning significantly improved during the pandemic. Interestingly, the change in family functioning was the strongest factor associated with the change in family happiness.

## Data availability statement

The original contributions presented in the study are included in the article/supplementary material, further inquiries can be directed to the corresponding author.

## Ethics statement

The studies involving human participants were reviewed and approved by the Ethics Committee of Ramathibodi Hospital, Mahidol University. The patients/participants provided their written informed consent to participate in this study.

## Author contributions

NL designed, conducted the study, mainly drafted, and finalized the manuscript. TP designed the study, performed statistical analysis, and wrote some sections of the manuscript. PW performed data collection and statistical analysis. All authors contributed to the article and approved the submitted version.
